# Post-Buckling Response of Carbon/Epoxy Laminates with Delamination under Quasi-Static Compression: Experiments and Numerical Simulations

**DOI:** 10.3390/ma17205047

**Published:** 2024-10-15

**Authors:** Fei Xia, Zikun Wang, Yi Wang, Heqing Liu, Jianghong Xue

**Affiliations:** 1 School of Civil Engineering, Henan Polytechnic University, Jiaozuo 454003, China; 2 School of Mechanics and Construction Engineering, Jinan University, Guangzhou 510632, China

**Keywords:** composites, delamination, post-buckling behavior, quasi-static compression

## Abstract

Delamination is a common type of damage in composite laminates that can significantly affect the integrity and stability of structural components. This study investigates the post-buckling behavior of carbon fiber-reinforced epoxy composite laminates with embedded delamination under quasi-static compression. Experimental tests were conducted using an electronic universal material testing machine to measure deformation and load-bearing capacity in the post-buckling stage. The specimens, prepared from T300 carbon fiber and TDE-85 epoxy resin prepreg, were subjected to axial compressive loads with delamination simulated by embedding Teflon films. Finite element analysis (FEA) was performed using ABAQUS software, incorporating a four-part model to simulate delaminated structures, with results validated against experimental data through comprehensive convergence analysis. The findings reveal that increasing delamination depth and length decrease overall stiffness, leading to an earlier onset of buckling. Structural instability was observed to vary with the size of delamination, while the post-buckling deformation mode consistently exhibited a half-wave pattern. This research underscores the critical impact of delamination on the structural integrity and load-bearing performance of composite laminates, providing essential insights for developing more effective design strategies and reliability assessments in engineering applications.

## 1. Introduction

Carbon fiber-reinforced epoxy composite laminates are extensively used in engineering applications, such as aerospace, automotive, vessel, etc., due to their remarkable mechanical properties and lightweight characteristics [1]. Composite components, often characterized as thin-walled structures, necessitate a crucial focus on ensuring their load-bearing stability throughout usage. However, during the manufacturing, processing, transportation and service processes, laminated composite material often encounter complex load effects, leading to unpredictable initial internal damage such as delamination [2], which significantly affects the stability and safety of the structure. Therefore, the post-buckling behavior analysis of laminated plates with delamination under quasi-static compression is crucial for evaluating reliability and predicting failure modes accurately.

The post-buckling performance of composite laminated structures under static loading has been studied by many scholars. Lu et al. [3] investigated the post-buckling behavior of carbon fiber-reinforced thermoplastic composite laminated plates using experimental methods, while further analysis of this phenomenon was conducted numerically [4]. Lal and Markad [5] presented a finite element model of laminated composite beams reinforced with multi-walled carbon nanotubes under varying thermal and mechanical loads for post-buckling analysis. Gao et al. [6] studied the impact of increasing thickness on the compressive properties of ultra-thick laminates, considering factors such as fiber volume fraction, fiber waviness, void content, and free edge effect. Mitrofanov and Shkurin [7] proposed a procedure to determine the thickness of thin load-bearing anisotropic panels under compressive and shear forces, providing analytical solutions for geometrically nonlinear problems and identifying potentially critical points. More literature on the post-buckling analysis of composite structures under axial compression include functionally graded carbon nanotubes [8], corrugated sandwich panels [9], hat-stiffened composite panels [10], and so on.

Delamination is a critical concern affecting the mechanical behavior and structural integrity of composite materials, particularly carbon fiber-reinforced polymers (CFRPs), across various loading conditions. Cheng et al. [11] examined the mechanical performance and damage behavior of delaminated composite laminates under different loading modes, providing insights into the effects of delamination on structural integrity. Li et al. [12] studied the effect of delamination defects on the mechanical properties of carbon fiber-reinforced polymer (CFRP) composites with different delamination areas, delamination depths, and delamination front shapes under compression. Kopparthi et al. [13] examined the failure behavior of delaminated carbon/epoxy composites under pure bending with additional failure modes explored in a separate work [14]. Bulut et al. [15] conducted double-cantilever beam and end-notched flexure tests to evaluate the delamination toughness properties of OP-filled composites. Furthermore, Cai et al. [16] revealed the influence of fabric stacking patterns and laying angles on the mechanical properties and failure mechanisms of composite laminates, employing finite element simulations to elucidate the relationship between laying configurations and structural performance. Bahrami et al. [17] investigated delamination formation in composite laminates subjected to bending loads, and the strain energy release rate was computed to detect dominant failure modes. Riccio et al. [18] and Nagendranath et al. [19] analyzed the buckling behavior of composite structures in aerospace applications. In terms of the thermal performance, Wang et al. [20] analyzed the influence of temperature-dependent characteristics of CFRP mechanical properties; Nikrad et al. [21] studied the nonlinear thermal post-buckling of functionally graded graphene-reinforced composite laminated plates with circular or elliptical delamination.

In the study of the mechanical properties of delaminated composite laminates, experimental methods could yield the actual response of structures, while numerical simulations could analyze the influence law of delamination defects. Aslan and Şahin [22] explored the buckling behavior and compressive failure of composite laminates containing multiple large delaminations, highlighting the significant impact of delamination on mechanical performance. Hu et al. [23] investigated the effect of initial delamination defects on L-stiffened composite panels under uniaxial compression using experiments and numerical simulations. They employed a progressive damage model and cohesive zone model to predict damage evolution, emphasizing the significant influence of delamination on structural performance. Mekonnen et al. [24] highlighted the effects of initial delamination size on post-buckling and delamination propagation in laminated composites subjected to axial compression, also addressing the influence of initial delamination location [25]. In the same way, Li et al. [26] revealed the detrimental impact of delamination on compressive strength and failure modes through experimental investigation.

In the matter of buckle response, Köllner [27] proposed a novel discrete coordinate approach for modeling nonlinear structural instability problems with material damage, while the interaction of delamination buckling and damage growth in cross-ply laminates was further discussed [28]. Muc [29] investigated the buckling phenomena and delamination mechanisms of laminates and functionally graded materials. Kosztowny and Waas [30] investigated the post-buckling response of unitized stiffened textile composite panels through experimental methods, which were complemented by computational modeling in a subsequent study [31]. Additionally, Shabanijafroudi and Ganesan [32] proposed a methodology to study buckling, post-buckling, and delamination growth, integrating analytical modeling with experimental validation. Ameri et al. [33] employed a cohesive zone model to investigate nonlinear post-buckling delamination in curved laminated composite panels. Calvo et al. [34] developed a modeling approach based on digital image correlation analysis to predict delamination failure under compressive loads in CFRP laminates.

Delamination defects significantly impact the post-buckling performance of laminated composite panels, making it crucial to understand their effects on structural integrity. The existing literature has explored various aspects of delamination, including its mechanical behavior and failure modes. However, there are still areas that require further investigation, particularly regarding the deformation modes of delaminated composite laminates—whether the buckling modes of delaminated laminated panels exhibit local buckling, global buckling, or mixed mode buckling.

The effects of delamination on the buckling behavior and deformation modes of laminated composite panels have been explored in our previous work [35]. The current study aims to further explore how delamination affects the post-buckling behavior of laminated composite panels. Quasi-static compression tests are conducted to assess the deformation and bearing capacity of structures in the post-buckling stage. Additionally, finite element simulations are performed using ABAQUS software (Version 6.14.4), enabling a detailed discussion of the parameterized mechanisms by which delamination damage impacts laminated structures. The results from these simulations are validated through comprehensive convergence analyses, aligning with experimental findings. This study seeks to provide a thorough investigation into the influence of delamination on post-buckling behavior, enhancing the understanding of failure mechanisms and contributing to the development of more reliable composite structures.

## 2. Research Object

**Figure 1:**
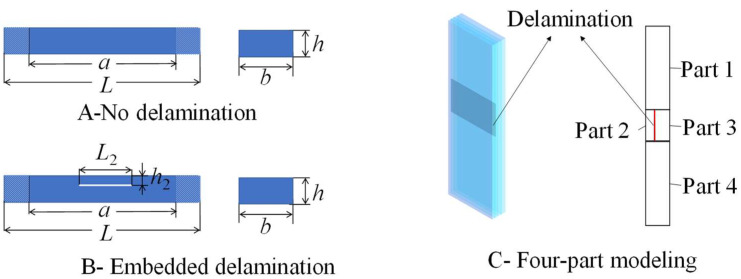
Laminated specimen in experiments and numerical simulations.

Consider narrow strips of carbon/epoxy composite with multiple layers. The laminated specimen has geometric dimensions of *L* × *b* × *h*, where *h* is the total thickness, and each single layer has a thickness of *h*_0_. The effective calculating length is *a*. The position and size of the initial delamination detect can be adjusted with the delamination having a length of *L*_2_ and a depth of *h*_2_. A four-part model is applied to simulate the laminated specimen in the finite element analysis, as shown in Figure 1.

The composite laminated specimen is formed by bonding multiple layers in a specific laying sequence, resulting in overall material performance that reflects anisotropy. The complex anisotropic relationship between the external loads and deformations of the composite laminated specimen is detailed in Appendix A.

**Figure 2:**
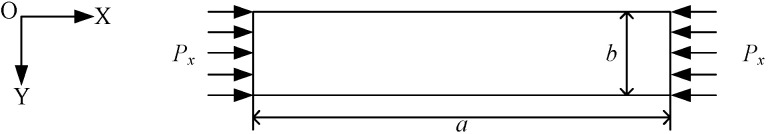
In-plane compressive load application diagram.

The composite specimen is subjected to an axial compressive load *P_x_* with clamped boundary conditions on both sides, as illustrated in Figure 2. In this specific implementation, the general post-buckling equilibrium path of laminated plate specimens is provided by the following [36].

(1) $$P_{x}=P_{cr}+P_{2i}\cdot w^{2}$$

where *w* is the maximum deflection of the composite laminate, *P_cr_* represents the critical buckling load and *P*_2*i*_ denotes the second-order load increment during the post-buckling deformation stage. Equation (1) outlines the load-bearing characteristics of the composite laminate in the post-buckling stage. Specifically, before failure occurs in the compressed laminate, the transverse deflection is directly related to the in-plane compressive load it sustains. Therefore, both the maximum transverse deflection and the load values of the composite laminated specimen during the quasi-static compression process must be obtained simultaneously to facilitate the experimental acquisition of the post-buckling equilibrium path.

## 3. Experiment

**Figure 3:**
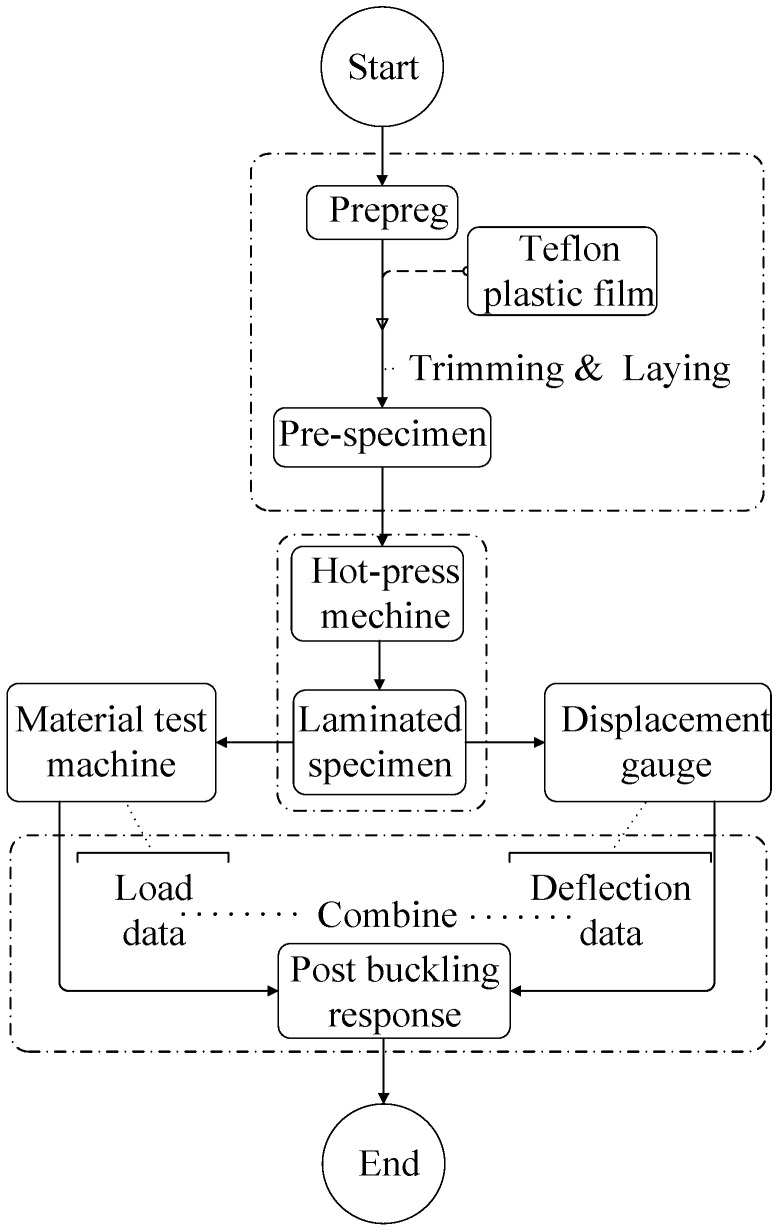
The flow chart of experimental procedure.

The post-buckling response of composite laminates with embedded delamination was investigated during the axial compression process using an electronic universal material test machine. As illustrated in Figure 3, the flow chart outlines the implementation process of the experiments conducted in this study.

The composite laminated specimens were prepared under hot-press loading by trimming and laying the single-layer prepreg previously. Unidirectional carbon fiber (T300)-reinforced epoxy resin (TDE-85) prepreg was utilized with the following parameters: *h*_0_ = 0.138 mm, *E*_11_ = 134 GPa, *E*_22_ = 10.2 GPa, and *G*_12_ = 5.52 GPa, *v*_12_ = 0.3. Each specimen had the same layup sequence and number of layers, represented as [0°/90°/0°]_6_, and were grouped into sets of 3–5 to minimize errors. To simulate the initial defect, a Teflon plastic film was placed at the initial delamination position. The total length of each specimen (*L*) was 250 mm. Table 1 lists the specific geometric parameters of the composite laminate specimens, where the numbers represent the depth and length of the initial delamination for both the experimental (E) and numerical simulation (F) specimens. For example, specimen E43 indicates that the delamination length is 40 mm with the delamination depth located at the third layer. Specimen F43 corresponds to the finite element model that incorporates the delamination parameters. Notably, specimen E00 represents a laminate without any delamination. It is important to emphasize that the geometric dimensions of the specimens are based on measurements taken after lamination, while the geometric parameters of delamination reflect the predetermined sizes established prior to lamination, specifically corresponding to the dimensions of the cut plastic film.

The specimens were reinforced at both ends by gluing aluminum sheets to ensure sufficient clamping strength. This resulted in an effective specimen length (*a*) of 170 mm. To obtain the load–deflection equilibrium path during the compression process, the maximum deflection deformation of the panel in the post-buckling stage was measured anyway. The maximum deflection deformation was typically selected as the deflection value in the middle region. Therefore, each specimen was marked along the midline to record deflection changes, representing the maximum deflection deformation.

**Figure 4:**
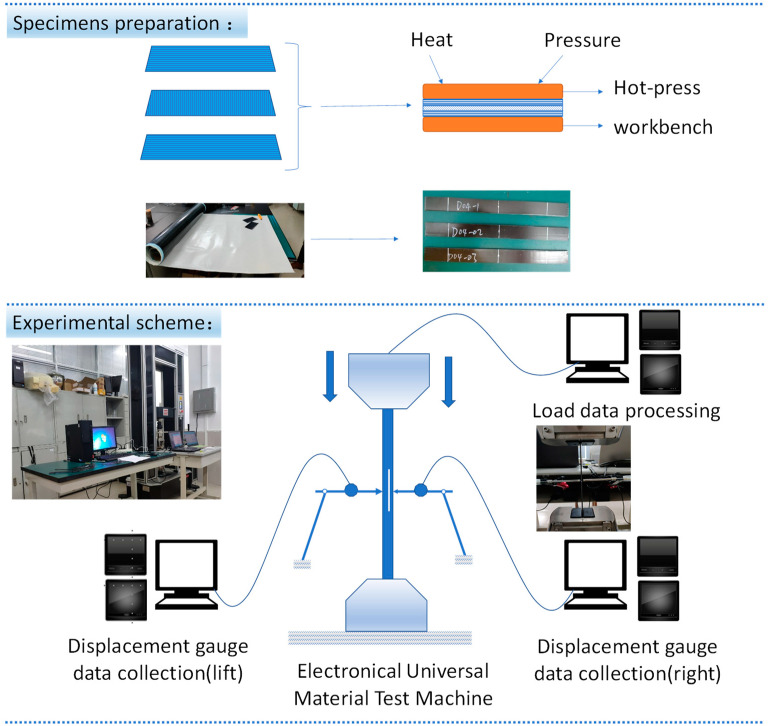
Experimental specimen and arrangement in this study.

To capture the post-buckling response of the composite laminate structure, axial compression tests were conducted, and data on in-plane loads and deformation were collected throughout the entire loading process. The compressive tests were performed in accordance with the test plan for the compressive performance of fiber-reinforced plastic thin layer panels within the testing machine system. Given the uncertainty in the post-buckling deformation direction of laminated panels after buckling bifurcation, two sets of displacement meter acquisition systems were established, as shown in Figure 4, which were denoted as displacement left and displacement right, respectively. Data collected under actual bending conditions during the process were considered valid results. The compressive tests were conducted using an electromechanical testing rig with a loading frequency of 5 Hz. In the data post-processing stage, the post-buckling curve was obtained by matching the two sets of data corresponding to load and deflection deformation.

## 4. Finite Element Analysis

Finite element simulations of the post-buckling response of test specimens were conducted using ABAQUS software (Version 6.14.4, License Number 27011) to verify the test solutions and facilitate an in-depth discussion. The FEA model was established using a three-dimensional deformable planar shell. To simulate delamination damage, four sub-laminated plates were constructed to represent the delaminated laminated panel specimen, as shown in Figure 1. Assembly and interaction constraints were systematically applied with the “hard” contact effect specifically included to analyze the interface properties of the prefabricated delamination areas. The material and geometric parameters used in the finite element model were identical to those of the test specimens and are provided in Table 1.

The FEA model was divided into 5000 quadrilateral elements using the Standard Linear Shell Element (S4R). The minimum element size was set to 0.001. Both the upper and lower ends of the model were constrained with fixed supports; the lower end was completely fixed, while the upper end was subjected to an axial compressive load. The significance of the post-buckling curve lies in studying the maximum deflection deformation corresponding to the pressure on the supporting section, which indicates the load-carrying ability under nonlinear deformation.

To achieve this, node sets for force and displacement were defined separately. The displacement node sets were selected from the central area of the model, typically the location of the maximum deflection deformation. Upon completion of the ABAQUS job, the load-carrying capacity in the post-buckling stage was determined by calculating the preload and load proportionality factor (LPF) from the history output during post-processing.

**Figure 5:**
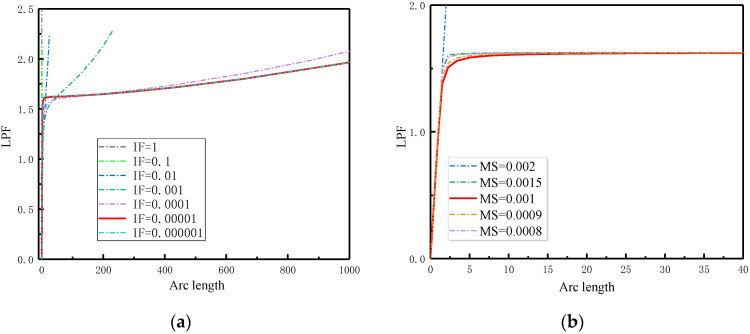
Convergence of FEA results of composite laminates specimen for different initial imperfection factors and mesh sizes. (**a**) Imperfection factor (IF); (**b**) mesh size (MS).

In the analysis of the post-buckling response of composite laminates subjected to in-plane compressive loads, the nonlinear effects due to large deformations were simulated by enabling geometric nonlinearity options. Initial geometrical imperfections were assigned to the model, matching the eigenvalue buckling mode, with the value of the imperfection being a proportionality factor of this mode. The determination of the imperfection value strategy depends on the convergence analysis of the model, as illustrated in Figure 5.

It can be concluded that the smaller the value of the imperfection factor (IF) and mesh size (MS) assumed, the more pronounced the characteristics of the post-buckling curve, although this increases the computation time. Therefore, values of 0.00001 for the imperfection factor and 0.001 for the mesh size were chosen for the FEA model in subsequent simulations to ensure both accuracy and efficiency.

## 5. Results and Discussion

The convergence of the finite element simulation model has been verified in the previous section by filtering the results of different imperfections and mesh sizes. In this section, a parametric study of the post-buckling response of the composite laminate structure was conducted by comparing the experimental and simulation results to reconfirm the validity of the FEA analysis. Then, the influence of delamination defects on the nonlinear deformation and load capacity of the composite laminate structure was thoroughly investigated. Additionally, the post-buckling deformation mode of the composite laminate structure was studied. Notably, the dimension of the axial compressive load *P_x_* is uniformly adopted from experimental conditions, i.e., N rather than N/m. Therefore, the linear load applied to the shell in the finite element model is converted to the appropriate units.

### 5.1. Experimental and FEM Results

**Figure 6:**
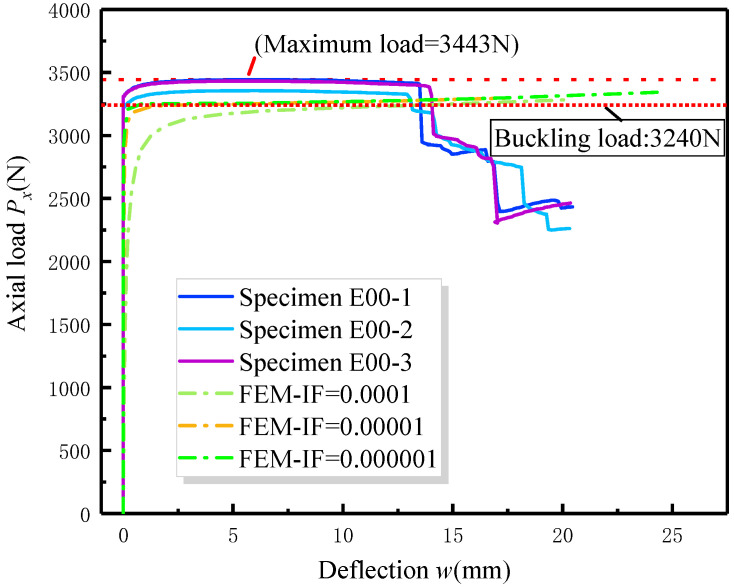
Comparison between the post-buckling curves obtained from the experiment and FEM for the A—No delamination specimen.

The post-buckling curves for composite laminated specimens, both with and without delamination, were obtained through experiments. Numerical simulations were also conducted under the same delamination conditions. Figure 6 presents three groups of post-buckling curves for specimens without delamination (A—No delamination), all of the same size, obtained from both test machines and finite element analysis (FEA) models. The finite element data were simulated by assuming three different values of initial imperfection, while the buckling load values from the finite element analysis are indicated as eigenvalue buckling lines in the diagram.

It is observed from Figure 6 that the three methods predict a similar post-buckling curve for the laminated specimen, and the out-of-plane deflection of the laminated panel grows gradually after the eigenvalue buckling stage. Despite this, the laminated structure did not experience collapse even after transverse deflection deformation had occurred. The maximum post-buckling load from the experimental results increased by 3.5–6.3% compared to the buckling load from the finite element simulation, indicating that the laminated specimen exhibits a certain degree of post-buckling strengthening.

As it should be, the increase in load capacity during the post-buckling stage is not significant for long and narrow strip structures. Additionally, the experimental results are generally higher than those from the finite element analysis. This discrepancy arises because factors such as initial imperfections are uncontrollable in experimental conditions compared to the simulation environment. Although smaller imperfections are assumed in the FEA simulation, experimental errors can significantly alter the results, including variations in specimen size, fixture misalignment, and geometric imperfections.

Overall, the buckling load and post-buckling strengthening curve of the laminated specimen obtained from experiments and finite element simulations are generally consistent, confirming the validity of both methods. Based on this, the following discussion will focus on the influence of delamination size on the buckling and post-buckling response of the laminated specimen.

**Figure 7:**
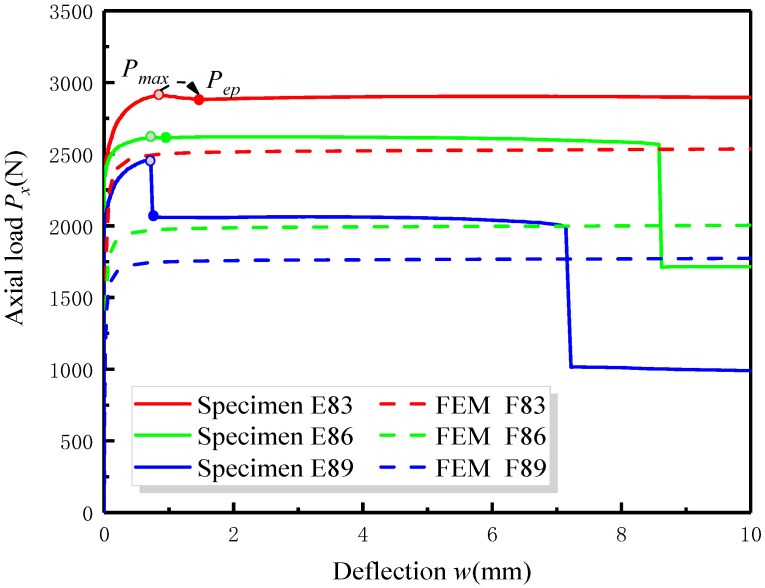
Comparison between the post-buckling curves obtained from the experiment and FEM for B—embedded delamination specimen with different delamination depth.

**Figure 8:**
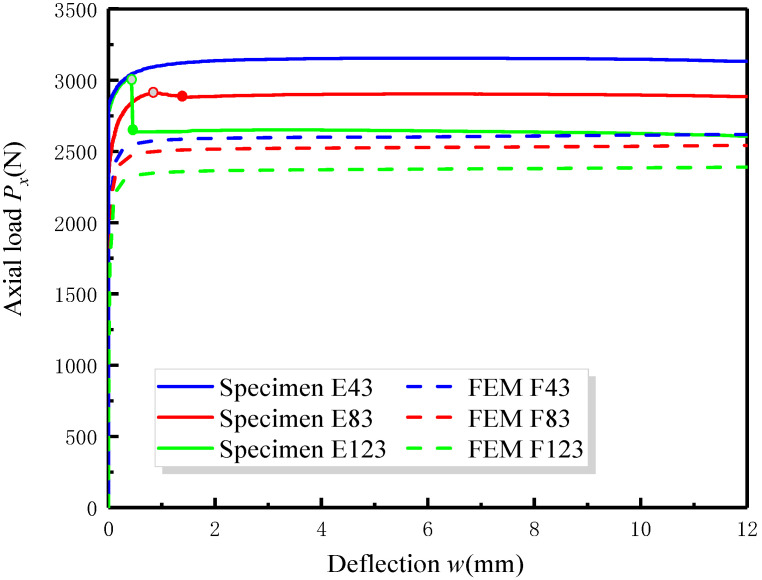
Comparison between the post-buckling curves obtained from the experiment and FEM for B—embedded delamination specimens with different delamination length.

Figure 7 is a schematic diagram showing the influence of delamination depth *h*_2_ on the post-buckling curve of specimens (B—embedded delamination) with the same delamination length *L*_2_. Figure 8 illustrates the post-buckling curve of specimens (B—embedded delamination) obtained from both experimental and FEA analyses under different delamination lengths *L*_2_. It is observed from Figure 7 and Figure 8 that the trends of the post-buckling curves under different delamination conditions exhibit similar changes in both experimental and finite element analyses.

For laminated specimens with a fixed delamination length, the buckling load and post-buckling load decrease with increasing delamination depth. Similarly, the buckling load and post-buckling load decrease with increasing delamination length for specimens with the same delamination depth. This indicates that the buckling and post-buckling loads are very sensitive to changes in delamination conditions. The greater the delamination depth and length, the smaller the overall stiffness of the laminated specimen, making it more prone to instability.

By comparing Figure 6, Figure 7 and Figure 8, a notable observation can be discovered. Under the same loading configuration, the load-bearing capacity of specimens with delamination does not continuously increase during the post-buckling stage. Instead, after reaching the maximum load, the post-buckling load curve exhibits a slight decline, as indicated in the section marked for specimen E83. This phenomenon is discussed in the analytical model of Köllner [27], where the delaminated laminate structure undergoes a transition to another equilibrium path during post-buckling deformation, which is also known as structural instability. To investigate this phenomenon more intuitively, Table 2 lists the maximum load *P_max_* and the equilibrium path load *P_ep_* after the transition for specimens with different delamination configurations. From Figure 7 and Figure 8 and Table 2, it can be seen that this transition in load values during the post-buckling stage shows minimal amplitude for the specimens E83, E86 and E43; however, for larger delamination lengths and depths, such as E89 and E123, the downward trend in the load curve is more pronounced. This may be due to the fact that larger delamination lengths and depths lead to a more significant reduction in the bending stiffness of the laminate, leading to a greater likelihood of unstable transitions. For all specimens, after the initial transition, the post-buckling curve maintains a long and smooth trend before final failure occurs.

### 5.2. Post-Buckling Deformation Mode

**Figure 9:**
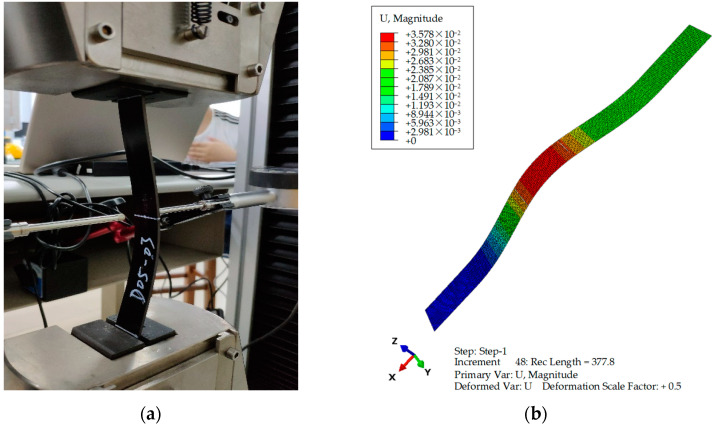
Comparison between the post-buckling deformation obtained from the experiment and FEM for B—embedded delamination specimen. (**a**) Experimental specimen E43; (**b**) FEM model F43.

To elaborate on the deflection deformation in the post-buckling stage of delaminated composite specimens, Figure 9 show the post-buckling deformation from both experimental and finite element simulation results, using the specimen E43/F43 from Table 1. As seen in Figure 9, the experimental and simulation research on post-buckling deformation is completely identical and reliable. A comparison between Figure 9a,b clearly demonstrates that the deflection deformation of delaminated composite specimens under in-plane compressive load, whether from experiment or FEA analysis, exhibits a globally consistent deformation with a half-wave mode. The theoretical models in Köllner [28] and Xia [35] also indicate similar global buckling modes for the delaminated laminates.

### 5.3. Influence of Delamination Size

**Figure 10:**
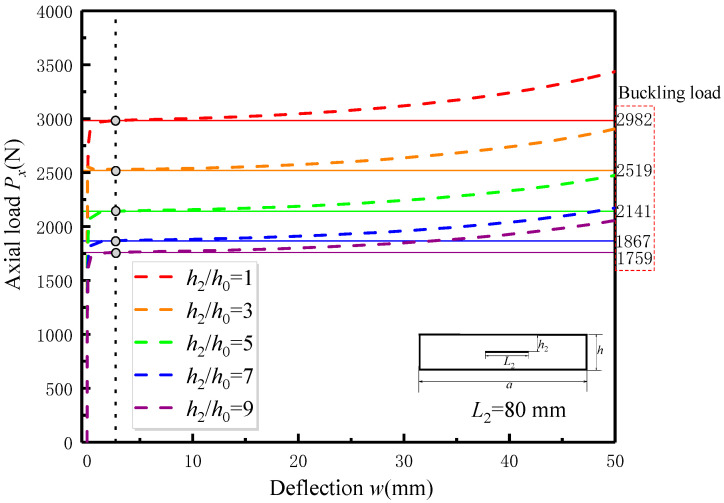
The influence of the delamination depth on post-buckling curve of delaminated specimen obtained from FEA analysis.

**Figure 11:**
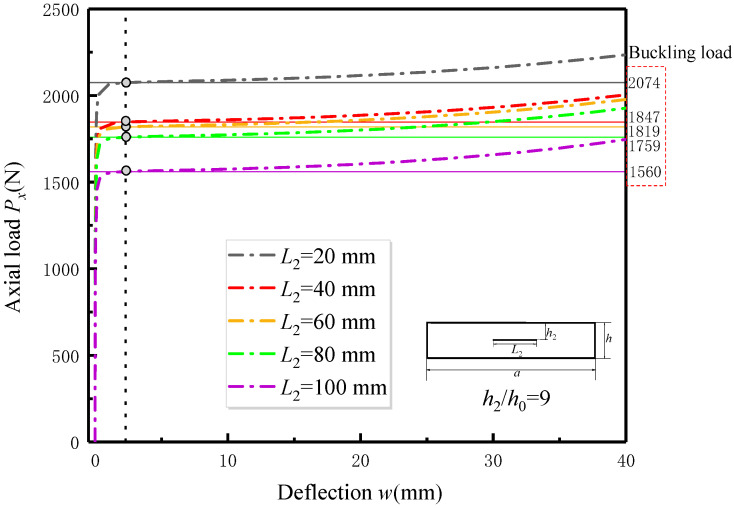
The influence of the delamination length on post-buckling curve of delaminated specimen obtained from FEA analysis.

After comparing and verifying the experimental results, further analysis and discussion were conducted on the FEA simulation results. Figure 10 illustrates the post-buckling curves of the delaminated specimens subjected to different delamination depths for a given delamination length (*L*_2_ = 80 mm). The post-buckling curves of the delaminated specimens with different delamination lengths for a given delamination depth (*h*_2_/*h*_0_ = 9) are shown in Figure 11. The influence of delamination size on the delaminated specimens is consistent with the conclusions obtained in the previous section.

As shown in Figure 10 and Figure 11, the eigenvalue buckling line and its intersection point with the post-buckling curve are highlighted accordingly. Different from nonlinear buckling theoretical analysis, out-of-plane deflection deformation already exists before eigenvalue buckling occurs in the post-buckling curve of delaminated specimens under in-plane compressive load calculated by the FEA model. Another phenomenon is that the values of deflection at the corresponding eigenvalue buckling loads are basically the same for all FEA models with different delamination sizes. This is because the initial geometric imperfection is presumed to be a proportional factor of the similar first-order buckling mode, which is not related to delamination defects.

## 6. Conclusions

This study investigated the post-buckling behavior of laminated composite structures with embedded delamination through experiments and numerical simulations under quasi-static compression. Experimental tests using electronic universal testing machine measured deformation and load in the post-buckling stage. Composite specimens with a [0°/90°/0°]_6_ configuration were prepared using unidirectional carbon fiber (T300)-reinforced epoxy resin (TDE-85) prepreg with delamination simulated by embedding PTFE films. Finite element analysis using ABAQUS, incorporating a four-part model, was performed to simulate the delaminated structure. The research primarily focused on the structural stability aspects of deformation and the load-bearing characteristics during the post-buckling stage. The following conclusions can be drawn:

1. Effect of delamination size on structural stability: As the delamination length and depth increased, the overall stiffness of the laminated specimens decreased, leading to a reduction in the post-buckling load-bearing capacity. As a result, the structural stability decreased, making the structure more prone to instability.
2. Post-buckling deformation mode: The post-buckling deformation of laminated composite specimens with delamination under in-plane compressive loads exhibited a globally consistent half-wave deformation mode. This is the primary deformation mode triggered in the structure, and the experimental results confirmed this observation.
3. Structural instability in post-buckling: By comparing the results, it was observed that the load-bearing capacity of delaminated specimens does not continuously increase during the post-buckling stage. Instead, after reaching the maximum load, the post-buckling load curve shows a slight decline, indicating a transition to another equilibrium path.
4. Effect of delamination on load transition: The transition in load values during the post-buckling stage varied with different delamination configurations. Specimens with smaller delamination exhibited minimal load changes after transition, while those with larger delamination showed a more pronounced downward trend in the load curve, which was likely due to the reduction in bending stiffness.
5. Post-buckling path and failure: After the initial transition, all specimens maintained a smooth post-buckling path. This trend suggests that delaminated laminated structures possess a certain load-bearing capacity during the post-buckling stage, sustaining it until failure occurs.

## 7. The Outlook

This study has provided valuable insights into the post-buckling behavior of laminated composite structures with embedded delamination. However, several limitations should be acknowledged. Firstly, the research focused exclusively on symmetric cross-ply laminates, which restricts the applicability of the findings to other laminate configurations. The complexities associated with asymmetric laminates, such as tension-bending and torsion-bending couple effects, were not explored.

Additionally, this study did not consider delamination growth during the post-buckling phase, which is a critical area of investigation. Future research should aim to incorporate this aspect, examining how delamination evolves under varying loading conditions and its subsequent effects on structural stability and performance.

Moreover, expanding the investigation to include other laminate stacking sequences and configurations would enhance understanding of post-buckling phenomena. Different layup orientations may exhibit distinct mechanical behaviors and failure mechanisms, providing a more comprehensive understanding of the structural integrity of laminated composites.

In summary, addressing these limitations and exploring the aforementioned areas will not only enrich the current knowledge base but also contribute to the development of more resilient composite materials in practical applications.

## Figures and Tables

**Table 1:** Geometric parameters of the specimens.

No.	Laminate Specimen		Delamination	
	Width *b* (mm)	Thickness *h* (mm)	Length *L*_2_ (mm)	Depth *h*_2_ (mm)
E00 (F00)	19.95/19.92/19.87	2.47/2.48/2.52	0	0
E43 (F43)	19.97/19.97/19.94	2.54/2.47/2.57	40	0.42
E83 (F83)	19.81/19.88/19.90	2.53/2.55/2.48	80	0.42
E123 (F123)	20.06/20.07/20.08	2.55/2.53/2.51	120	0.42
E86 (F86)	20.17/20.20/19.96	2.56/2.53/2.72	80	0.83
E89 (F89)	20.00/20.04/19.99	2.50/2.55/2.51	80	1.25
FEM (F)	20	2.484	-	-

**Table 2:** Maximum load (*P_max_*) and equilibrium path load (*P_ep_*) of the specimens.

No.	E43	E83	E123	E86	E89
*P_max_* (N)	3154	2910	3026	2616	2464
*P_ep_* (N)	-	2880	2635	2613	2058

## Data Availability

The original contributions presented in the study are included in the article, further inquiries can be directed to the corresponding author.

## Nomenclature

*L*	Total length of specimen (mm)	MS	Mesh size
*a*	Effective length of specimen (mm)	IF	Imperfection factor
*b*	Width of specimen (mm)	LPF	Load proportionality factor
*h*	Total thickness of specimen (mm)	E	Specimen for experiment
*θ*	Laying angle of specimen (mm)	F	Specimen for simulation
*h*_0_	Thickness of single-layer prepreg (mm)	S4R	Four-node reduced integration shell element
*L*_2_	Delamination length (mm)	*P_x_*	In-plane compressive load (N)
*h*_2_	Delamination depth (mm)	*P_cr_*	Critical buckling load (N)
*w*	Maximum deflection of specimen (mm)	*P*_2*i*_	Second-order load increment during post-buckling stage
*P_max_*	Maximum load of post-buckling stage (N)	*P_ep_*	Equilibrium path load of post-buckling stage (N)

## Appendix A

The constitutive relationship of the symmetric cross-ply laminates specimen is given by the following [35]:

(A1) $$\left[\begin{array}{c} \left[P\right]\\ \left[M\right] \end{array}\right]=\left[\begin{array}{cc} \left[A_{ij}\right] & 0\\ 0 & \left[D_{ij}\right] \end{array}\right]\left[\begin{array}{c} \left[\varepsilon \right]\\ \left[\kappa \right] \end{array}\right]$$

where [*P*], [*M*], [*ε*], and [*κ*] are the matrices of the membranous force, bending moments, in-plane strain, and curvature of the neutral plane, respectively; [*A_ij_*] and [*D_ij_*] represent the membranous stiffness and bending stiffness and are computed by the following formulas:

(A2) $$\left(\left[A_{ij}\right],\left[D_{ij}\right]\right)=\int_{-\frac{h}{2}}^{\frac{h}{2}}{\bar{Q}}_{ij}^{\left(k\right)}(1,z^{2})dz\;(i,j=1,2,6)$$

where ${\bar{Q}}_{ij}^{\left(k\right)}$ is the stiffness coefficient matrix of the *k*-th ply in the global coordinate system with the laying angle *θ*, and it is calculated by the following:

(A3) $$\left[\begin{array}{c} {\bar{Q}}_{11}^{\left(k\right)}\\ {\bar{Q}}_{12}^{\left(k\right)}\\ {\bar{Q}}_{22}^{\left(k\right)}\\ {\bar{Q}}_{16}^{\left(k\right)}\\ {\bar{Q}}_{26}^{\left(k\right)}\\ {\bar{Q}}_{66}^{\left(k\right)} \end{array}\right]=\left[\begin{array}{cccc} C^{4} & 2C^{2}S^{2} & S^{4} & 4C^{2}S^{2}\\ C^{2}S^{2} & C^{4}+S^{4} & C^{2}S^{2} & -4C^{2}S^{2}\\ S^{4} & 2C^{2}S^{2} & C^{4} & 4C^{2}S^{2}\\ C^{3}S & CS^{3}-C^{3}S & -CS^{3} & -2CS(C^{2}-S^{2})\\ CS^{3} & C^{3}S-CS^{3} & -C^{3}S & 2CS(C^{2}-S^{2})\\ C^{2}S^{2} & -2C^{2}S^{2} & C^{2}S^{2} & {\left(C^{2}-S^{2}\right)}^{2} \end{array}\right]\left[\begin{array}{c} Q_{11}\\ Q_{12}\\ Q_{22}\\ Q_{66} \end{array}\right]$$

where

(A4) $$\begin{array}{l} C=\cos\theta \;S=\sin\theta \;Q_{11}=E_{11}/\left(1-\mu_{12}\mu_{21}\right)\;Q_{22}=E_{22}/\left(1-\mu_{12}\mu_{21}\right)\\ Q_{12}=\mu_{12}Q_{22}=\mu_{21}Q_{11}\;Q_{66}=G_{12}\mu_{12}E_{22}=\mu_{21}E_{11} \end{array}$$

where *E*_11_, *E*_22_, *G*_12_, *μ*_12_ and *μ*_21_ are the engineering elastic parameters of single-layer material.

## References

[1] Goyal V.K., Pennington A. FEA-based failure analysis of post-buckled composite multi-hat stiffened panel under static compression. Proceedings of the American Society for Composites (ASC) 37th Annual Technical Conference.

[2] Huang T., Bobyr M. (2023). A review of delamination damage of composite materials. J. Compos. Sci..

[3] Lu T.Y., Shen H.S., Wang H., Chen X.H., Feng M.L. (2023). Nonlinear bending and thermal postbuckling of thermoplastic composite laminated plates under temperature variation. Thin-Walled Struct..

[4] Lu T.Y., Shen H.S., Wang H., Chen X.H. (2022). Compression-after-impact effect on postbuckling behavior of thermoplastic composite laminated plates. Aerosp. Sci. Technol..

[5] Lal A., Markad K. (2019). Thermo-mechanical post buckling analysis of multiwall carbon nanotube-reinforced composite laminated beam under elastic foundation. Curved Layer. Struct..

[6] Gao Y., Zhu S.Y., Ding H.M., Song X.W., Hu H.Y., Wang H., Ke Y.L. (2023). Thickness variation effect on compressive properties of ultra-thick CFRP laminates. Int. J. Mech. Sci..

[7] Mitrofanov O., Shkurin M. (2023). Design of load-bearing anisotropic wing box panels ensuring static strength in the post buckling state. Aerosp. Syst..

[8] Chan D.Q., Nguyen P.D., Quang V.D., Anh V.T., Duc N.D. (2019). Nonlinear buckling and post buckling of functionally graded CNTs reinforced composite truncated conical shells subjected to axial load. Steel Compos. Struct..

[9] Chen L.M., Peng S.W., Liu J., Liu H.C., Chen L.L., Du B., Li W.G., Fang D.N. (2020). Compressive response of multi-layered thermoplastic composite corrugated sandwich panels: Modelling and experiments. Compos. Part B Eng..

[10] Bouslama N., Maslouhi A., Masson P. (2022). Experimental and numerical investigation of damage evolution in carbon fiber reinforced polymer stiffened panel in post buckling regime. J. Compos. Mater..

[11] Cheng P., Peng Y., Wang K., Wang Y.Q. (2021). Mechanical performance and damage behavior of delaminated composite laminates subject to different modes of loading. J. Braz. Soc. Mech. Sci. Eng..

[12] Li Y.L., Wang B., Zhou L. (2023). Study on the effect of delamination defects on the mechanical properties of CFRP composites. Eng. Fail. Anal..

[13] Kopparthi P.K., Gemaraju S., Pathakokila B.R., Gamini S. (2021). Failure analysis of delaminated carbon/epoxy composite under pure bending: Validation with numerical analysis. Multidiscip. Model. Mater. Struct..

[14] Kopparthi P.K., Aerra KK Y., Pathakokila B.R., Gamini S. (2022). Bending and viscoelastic behaviour of delaminated woven E-glass/epoxy composite. Aust. J. Mech. Eng..

[15] Bulut M., Alsaadi M., Erkliğ A. (2022). Effect of olive pomace particles content on mode I and mode II delamination fracture of S-glass fiber reinforced composites. Polym. Compos..

[16] Cai Y., An X.Z., Zou Q.C., Fu H.T., Yang X.H., Zhang H. (2021). Mechanical properties and failure mechanisms of composite laminates with classical fabric stacking patterns. J. Mater. Sci..

[17] Bahrami M., Malakouti M., Farrokhabadi A. (2021). Anticipating the induced delamination formation in composite laminates subjected to bending loads. Fatigue Fract. Eng. Mater. Struct..

[18] Riccio A., Castaldo R., Palumbo C., Russo A. (2023). Delamination Effect on the Buckling Behaviour of Carbon–Epoxy Composite Typical Aeronautical Panels. Appl. Sci..

[19] Nagendranath A., Khalane S.A., Gupta R.K., Rao C.L. (2023). Delamination Buckling of Composite Conical Shells Under External Pressure. Def. Sci. J..

[20] Wang H.X., Wu Y.H., Zhang Y., Zhang X.H. (2023). Influence of the temperature-dependent characteristics of CFRP mechanical properties on the critical axial force of drilling delamination. Polymers.

[21] Nikrad S.F., Chen Z.T., Akbarzadeh A.H. (2023). Nonlinear thermal postbuckling of functionally graded graphene-reinforced composite laminated plates with circular or elliptical delamination. Acta Mech..

[22] Aslan Z., Şahin M. (2009). Buckling behavior and compressive failure of composite laminates containing multiple large delaminations. Compos. Struct..

[23] Hu C.X., Bai Y.J., Xu Z.H., Qiu J.Z., Wang R.G., He X.D. (2022). Progressive damage behavior of composite L-stiffened structures with initial delamination defects under uniaxial compression: Experimental and numerical investigations. Polym. Compos..

[24] Mekonnen A.A., Woo K., Kang M., Kim I.G. (2020). Effects of size and location of initial delamination on post buckling and delamination propagation behavior of laminated composites. Int. J. Aeronaut. Space Sci..

[25] Mekonnen A.A., Woo K., Kang M., Kim I.G. (2020). Post buckling and delamination propagation behavior of composite laminates with embedded delamination. J. Mech. Sci. Technol..

[26] Li H.H., Yao Y.T., Guo L.Y., Zhang Q.H., Wang B. (2018). The effects of delamination deficiencies on compressive mechanical properties of reinforced composite skin structures. Compos. Part B Eng..

[27] Köllner A., Wadee M.A. (2022). A novel discrete coordinate approach to modelling nonlinear structural instability problems with material damage. Eur. J. Mech. ASolids.

[28] Köllner A., Kashtalyan M., Guz I., Völlmecke C. (2020). On the interaction of delamination buckling and damage growth in cross-ply laminates. Int. J. Solids Struct..

[29] Muc A. (2022). Buckling of composite structures with delaminations—Laminates and functionally graded materials. Appl. Sci..

[30] Kosztowny CJ R., Waas A.M. (2021). Postbuckling response of unitized stiffened textile composite panels: Experiments. Int. J. Non-Linear Mech..

[31] Kosztowny CJ R., Waas A.M. (2021). Postbuckling response of unitized stiffened textile composite panels: Computational modeling. Int. J. Non-Linear Mech..

[32] Shabanijafroudi N., Ganesan R. (2021). A new methodology for buckling, postbuckling and delamination growth behavior of composite laminates with delamination. Compos. Struct..

[33] Ameri B., Moradi M., Mohammadi B., Salimimajd D. (2020). Investigation of nonlinear post buckling delamination in curved laminated composite panels via cohesive zone model. Thin-Walled Struct..

[34] Calvo J.V., Feito N., Miguélez M.H., Giner E. (2022). Modeling the delamination failure under compressive loads in CFRP laminates based on digital image correlation analysis. Compos. Struct..

[35] Xia F., Jian F., Zhu Z., Li P., Liang H., Jin F., Xue J. (2019). A modified first order shear deformation theory for Reissner-Mindlin composite panels with internal delamination. Compos. Struct..

[36] Xue J., Jin F., Zhang J., Li P., Xia F., Xu J., Liu R., Yuan H. (2019). Post buckling induced delamination propagation of composite laminates with bi-nonlinear properties and anti-penetrating interaction effects. Compos. Part B Eng..

